# News and Coming Events

**Published:** 1907-08-03

**Authors:** 


### NEWS AND COMING EVENTS.

Dr. Graham Steell, M.D., F.R.C.P., has been appointed to the Chair of Medicine at the University of Manchester.

---

Four boys between the ages of 14 and 17 were recently arrested in Newark for selling cocaine to schoolboys.

---

During the year 1906 no less than 584,632 patients, intern and extern, were treated in the Government dispensaries in the North-West Frontier Province, India.

---

The French Surgical Association will hold its 20th annual congress in Paris on October 7. M. Paul Berger, surgeon to the Hôpital Neckar, will preside over the deliberations of the conference.

---

The summer meeting and annual luncheon of the Irish Medical Schools' and Graduates' Association will this year be held at Exeter at the Rougoumont Hotel, on Thursday, August 1, at 1 p.m.

---

Professor Van Leyden, who has held the Chair of Special Pathology at the University of Berlin for close upon 30 years, has just resigned his appointment as lecturer, though he will still retain his posts in the clinic. To the vacancy has been appointed Professor Wilhelm His.

---

It has been decided to endow a bed in perpetuity at the Royal Maternity Hospital, Edinburgh, to be called the "Alexander Simpson Bed," in recognition of the services of Sir Alexander Simpson.

---

On the 19th ult. Mr. Percival Nairne, Chairman of the Seamen's Hospital, unveiled a portrait of the Hon. Bomanjii Petit, of Bombay, at the London School of Tropical Medicine, Victoria and Albert Docks. Mr. Petit has been keenly interested in the school, and has been one of its most generous supporters.

---

The late Professor Grancher, of Paris, whose death took place a few days ago, has set the seal on his lifelong efforts to combat the ravages of tuberculosis by a handsome legacy, producing some £800 a year, to the institution which he founded for the protection of children against this terrible scourge of civilisation. This institution, which is under the direction of Dr. Roux, the President of the Pasteur Institute, provides for the removal of healthy children from poor families where tuberculosis has made its appearance to healthy country homes, in which, free from the chance of contagion, they may grow up under the charge of kindly peasants in clean air and under conditions favourable to normal development.

